# Real-time detection of neural oscillation bursts allows behaviourally relevant neurofeedback

**DOI:** 10.1038/s42003-020-0801-z

**Published:** 2020-02-14

**Authors:** Golan Karvat, Artur Schneider, Mansour Alyahyay, Florian Steenbergen, Michael Tangermann, Ilka Diester

**Affiliations:** 1 Optophysiology - Optogenetics and Neurophysiology, Albert-Ludwigs-University, Albertstrasse 23, 79104 Freiburg, Germany; 2 Bernstein Center for Computational Neuroscience, Albert-Ludwigs-University, Hansastr. 9, 79104 Freiburg, Germany; 3 BrainLinks-BrainTools / Intelligent Machine-Brain Interfacing Technology (IMBIT), Albert-Ludwigs-University, Georges-Köhler-Allee 201, 79110 Freiburg, Germany; 4 Faculty of Biology III, Albert-Ludwigs-University, Schänzlestr. 1, 79104 Freiburg, Germany; 5 Brain State Decoding Lab, Albert-Ludwigs-University, Albertstrasse 23, 79104 Freiburg, Germany; 6 Department of Computer Science, Albert-Ludwigs-University, Georges-Köhler-Allee 080, 79110 Freiburg, Germany

**Keywords:** Brain-machine interface, Motor cortex

## Abstract

Neural oscillations as important information carrier in the brain, are increasingly interpreted as transient bursts rather than as sustained oscillations. Short (<150 ms) bursts of beta-waves (15–30 Hz) have been documented in humans, monkeys and mice. These events were correlated with memory, movement and perception, and were even suggested as the primary ingredient of all beta-band activity. However, a method to measure these short-lived events in real-time and to investigate their impact on behaviour is missing. Here we present a real-time data analysis system, capable to detect short narrowband bursts, and demonstrate its usefulness to increase the beta-band burst-rate in rats. This neurofeedback training induced changes in overall oscillatory power, and bursts could be decoded from the movement of the rats, thus enabling future investigation of the role of oscillatory bursts.

## Abstract (web summary)

Karvat et al. describe a real-time digital signal-processing method to detect neural oscillations in the form of short and narrowband bursts. Using this real-time method, they show that neurofeedback increases burst-related behaviours and that these bursts can be decoded from the movements of the rats.

## Introduction

Neural oscillations are a frequently reported indicator of neural activity measured invasively via extracellular recordings as local field potentials (LFP), or non-invasively by magnetoencephalogram (MEG) or electroencephalogram (EEG)^1^. In recent years, neural oscillations are increasingly interpreted as transient bursts rather than sustained oscillations^2–5^, and bursts were even suggested as the primary ingredient of all band-specific activity^6^. These transient events appear in physiologically relevant time windows^1^, which makes them optimal candidates to shape behaviour in a trial-by-trial fashion^7^. Despite the increasing attention to these transient bursts, their role in neural computation, and ultimately in producing behavioural outputs, remains controversial^3^.

If indeed bursts of neural oscillations play a role in behaviour, we hypothesize that (1) reinforcing bursts would increase the occurrence of the burst-related behaviour and thus burst-rate (i.e., neurofeedback), (2) burst-rate-increase will lead to global (averaged over long periods) power increase, and (3) burst occurrences can be predicted based on behavioural readouts.

As a testbed for these hypotheses, we chose the rat motor cortex and focused on LFP bursts of beta-waves (15–30 Hz), which have been documented in humans, monkeys and mice. These short bursts (<150 ms) have been correlated with memory, movement and perception^4–6,8,9^, and reported to be correlated with behaviour up to 300 ms relative to the burst occurrence^8^.

Although changes in beta-power were detected online in previous work^10^, a method to measure and identify these narrow-band and short-lived bursts in real-time for addressing the abovementioned hypotheses is missing. The first challenge in developing such a method is to formally define LFP bursts. We suggest defining a burst as a power peak in time and frequency, exceeding a threshold^5^. When defining the threshold, two key points have to be addressed: first, it should be calculated from the statistics of the ongoing LFP recording, as the global LFP-power can change between subjects, between and even over sessions. Second, it should be based on a defined percentile, as opposed to central tendency measures (i.e. mean and median), to assure a statistically sound significance definition under non-normal distributions.

The second challenge is the detection of such short-lived peaks, which requires minimal pre-processing and delay, as well as high time and frequency resolutions. Here we present a real-time digital signal processing (DSP) method, capable to detect short- and narrow-band bursts. We demonstrate the utility of the system by reinforcing beta-bursts for neurofeedback training, which induced changes in overall beta-power. Further, we provide evidence that bursts can be decoded from the movement of the rats, thus enabling future investigation of the role of oscillatory bursts in behaviour.

## Results

### β-bursts can be detected in real-time

**Fig. 1:**
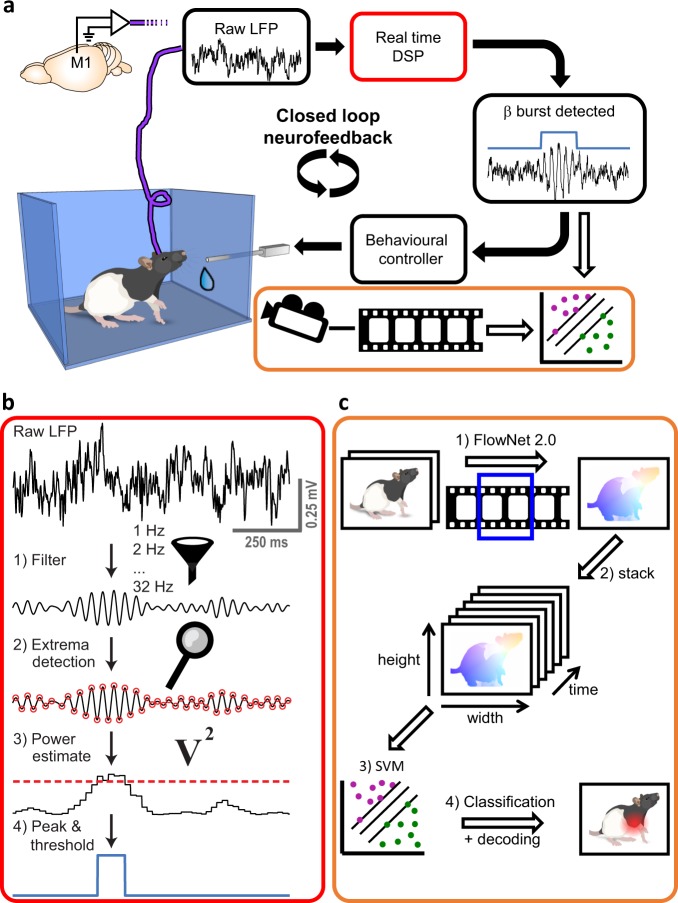
Overview of LFP β-event based neurofeedback method. **a** The Setup. LFP signals from the motor cortex of a freely moving rat were measured and fed into the real-time digital signal processing unit (DSP, red outline). Upon detection of an LFP beta-burst, the rat was rewarded with sucrose water. The activity of the rat was videotaped in synchronization with the electrophysiological data, and videos were analysed offline by a machine learning algorithm to detect movements indicative of beta bursts (orange outline). Black arrows: online analysis. White arrows: offline analysis. **b** Real-time LFP-burst detection algorithm. (1) The raw signal was filtered by an array of digital narrow-band finite-impulse response filters. (2) Turning points were detected in the filtered signal. (3) The square of the amplitude in a turning point was latched until the detection of the next extrema and served as an estimate of power. (4) If the power in a specific frequency was higher than the power of the frequency above and the frequency below, as well as the value of the 98th percentile of the target frequency power calculated online, it was defined as a burst. A burst was rewarded if it happened in the targeted frequencies, and lasted ≥ 70 ms. **c** Offline algorithm for decoding behaviour. A support vector machine (SVM) model was trained to classify epochs with or without beta bursts (as detected by the real-time DSP) based on behaviour. Movements of the rat (1) were approximated via optical flow, calculated from adjacent frames with FlowNet 2.0^23^. A stack of flow images (2) was used as an input for the SVM classifier (3). Classification accuracy and attention (defined as the distance to the decision function) were used to evaluate the model in the temporal and spatial domains (4). Rat images in a and c: Copyright (c) 2015 Etienne Ackerman, modified and published with permission under the MIT License (MIT). Brain image in a: Copyright (c) Wenbo Tang, modified and published with permission under SciDraw license.

**Fig. 2:**
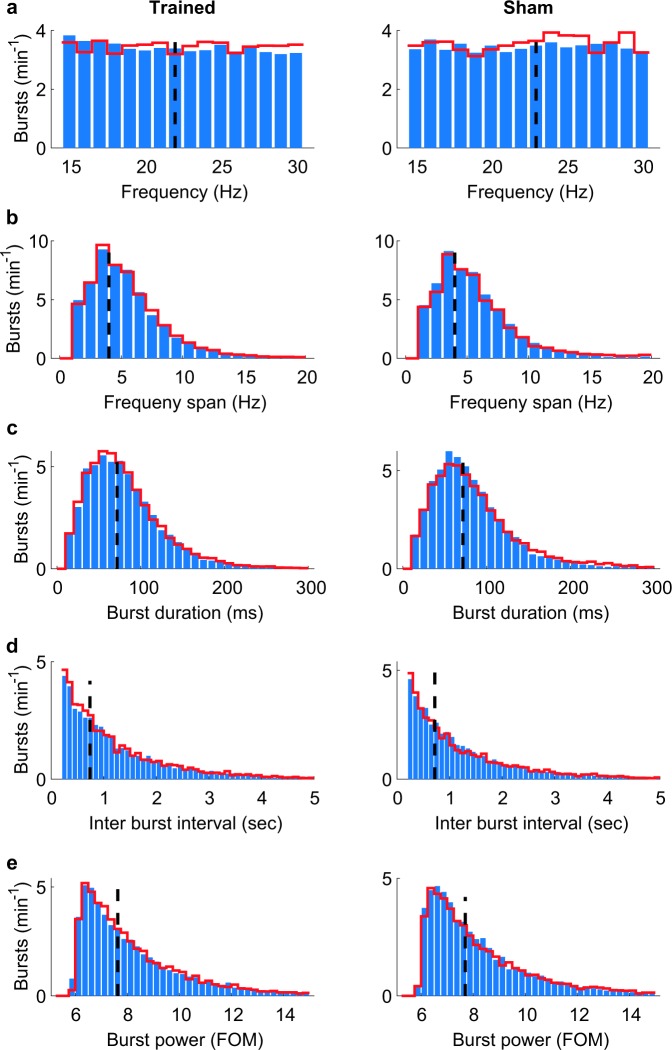
Offline characterization of beta-band bursts. Bursts were detected offline as peaks in time and frequency of the wavelet time-frequency representation (similar to a previous study^8^), which exceed the 98th percentile^5^ in the first three sessions (blue bars) and last three sessions (red lines) of rats trained in the neurofeedback paradigm (left panels) or sham-trained rats (right panels). Dashed black lines indicate the median. **a** Peak frequency. **b** Frequency span, defined as the distance (in Hz) between the lowest and highest frequencies with power above the 98th percentile at the peak time-point. **c** Burst duration, defined as the distance (in ms) between the first and last time points with power above the 98th percentile of the peak frequency. **d** Inter-burst-interval. **e** Burst power, presented as the fraction of the median (FOM).

A system is formally considered to perform in real-time if it responds within a guaranteed time constraint^11^. In addition, the system is required to have a sufficiently short delay to effectively influence its environment^12^. Our DSP algorithm (Fig. 1) is designed to measure and identify narrow-band and short-lived (Fig. 2) bursts. It is based on 32 digital bandpass filters operated within an acquisition system with a guaranteed processing time. The finite-impulse-response (FIR) filters have a width (at half-magnitude) of 5 Hz, and are centred on steps of 1 Hz. The acquisition system detects peaks and troughs in the filtered data of each frequency online, and determines the power based on the amplitude of these extrema^13^. As both peaks and troughs are taken into account, the time resolution is half the period of each frequency. The linear phase characteristic of the FIR filters ensures that there is no distortion due to the time delay of frequencies relative to one another, resulting in a fixed delay of 130 ms for each frequency (see Methods and Supplementary Movie 1). The fixed delay allows a direct comparison of neighbouring frequencies necessary for peak detection (Supplementary Fig. 1). By directly comparing frequencies, we were able to optimize the trade-off between peak-frequency resolution (1 Hz) and temporal delay (130 ms, Supplementary Fig. 2), outperforming conventional online methods (Supplementary Fig. 3). Importantly, this fixed delay is sufficiently short to plausibly influence behaviour^8^, thus the system fulfils the formal conditions of a real-time system^11,12^. To close the loop between oscillatory events and behaviour, we linked the DSP system with an operant conditioning apparatus for rodents, and synchronized videos with the LFP-recordings for offline behavioural analysis (Fig. 1c).

### Neurofeedback increases β-burst-related behaviours and power

For demonstrating the efficacy of the real-time method and investigating whether rats can volitionally increase the occurrence of beta-burst-related behaviours and beta-power, we implanted laminar probes in the motor cortex of three rats. The freely moving rats were placed in the closed-loop neurofeedback apparatus, where nearly artefact-free LFP was measured and analysed in real-time. For neurofeedback training, occurrences of oscillatory bursts in one of the frequencies in the beta-band (20–25 Hz for 2 rats, 15–20 Hz for 1 rat), longer than 70 ms (median duration of all bursts as analysed offline, Fig. 2c) and higher than the 98th percentile of power (adopting a previously used value^5^) were rewarded (Supplementary Movie 1). The power threshold was dynamic and updated every second based on the preceding 15 s. These values ensured ~100 rewards per 30 min session, thus keeping the rats motivated. Hence, in early sessions animals were rewarded for spontaneously occurring beta bursts and had to learn over sessions to increase the occurrence and power of beta bursts. To test whether the approach works for different sub-bands, we targeted 20–25 Hz for two rats, and 15–20 Hz for one rat. To control for non-specific reward-related effects on neural activity, we conducted sham training on two additional rats. These rats were implanted with identical probes in M1, and were given water at the times at which trained rats were rewarded (regardless of their current brain activity), i.e. the reward history of the trained rats was replayed to the sham-control rats.

**Fig. 3:**
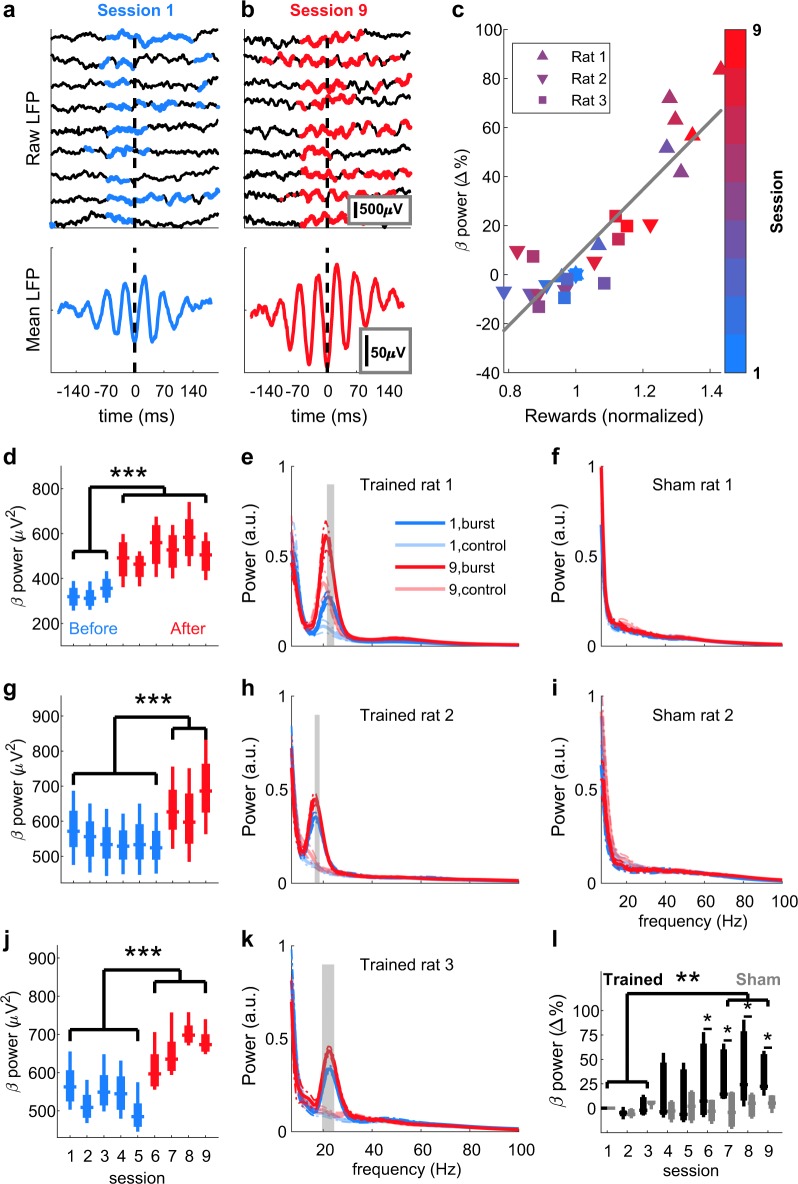
Neurofeedback increases β-burst power. Examples of raw LFP traces from the first (**a**, top) and last (**b**, top) of the nine neurofeedback training sessions. Time points in which beta-power exceeded the threshold defined as the 98th percentile are marked in blue (**a**) or red (**b**). Reward was delivered at time = 0. The heterogeneity of the individual bursts disappeared when averaging all bursts of one session, resulting in a sustained oscillation (**a**, **b**, bottom). **c** Correlation between number of rewards and session mean beta-power relative to session 1 for each rat in each session. Colours indicate the session number and each rat is denoted with a different shape. Pearson’s ρ = 0.89, *p* = 5.9 × 10^–10^. Mean power analysis of the targeted beta frequencies (20–25 Hz for rats 1 and 3 [**d** and **j**], 15–20 Hz for rat 2[**g**]) revealed for each rat a significant increase in power in a certain session (aha-moment) that persisted until the end of the experiment. Sessions before the aha-moment are represented in blue, and after the aha-moment in red. Two-way ANOVA (frequency and session), effect for session: rat 1: F(5,8) = 101.99, *p* = 3.81 × 10^−24^, rat 2: F(5,8) = 85.73, *p* = 10^−22^, rat 3: F(5,8) = 248.85, *p* = 1.29 × 10^−31^. ***p < 5.19 × 10^−6^, multiple comparisons with Bonferroni correction. Analysis of the broadband power 200 ms prior to reward delivery in a session before (blue) and after (red) neurofeedback training (**e**, **h**, **k**) or sham training (**f**, **i**) is plotted as mean ± 95% confidence interval (dashed). Faded lines: power 200 ms post reward delivery. Grey shading: frequencies in which the difference between power before and power after reward was significantly (*p* < 0.01) different after training compared to before training (*t*-test with Bonferroni correction). Digits (1 or 9) in the legend in **e** indicate the session numbers, “burst” refers to 200 ms prior to rewards and “control” to 200 ms after reward. **l** Group averaged beta-power change relative to the first session. Three-way ANOVA (rat, session and treatment), effect for session: trained: F(8,16) = 2.864, *p* = 0.0349, sham: F(8,8) = 0.385, *p* = 0.9. Effect for treatment: F(1,35) = 4.99, *p* = 0.032. **p < 0.01, *p < 0.05, multiple comparisons with Bonferroni correction. Presented elements in **d, g, j, l** centre line: median; box limits: upper and lower quartiles; whiskers: full distribution.

**Fig. 4:**
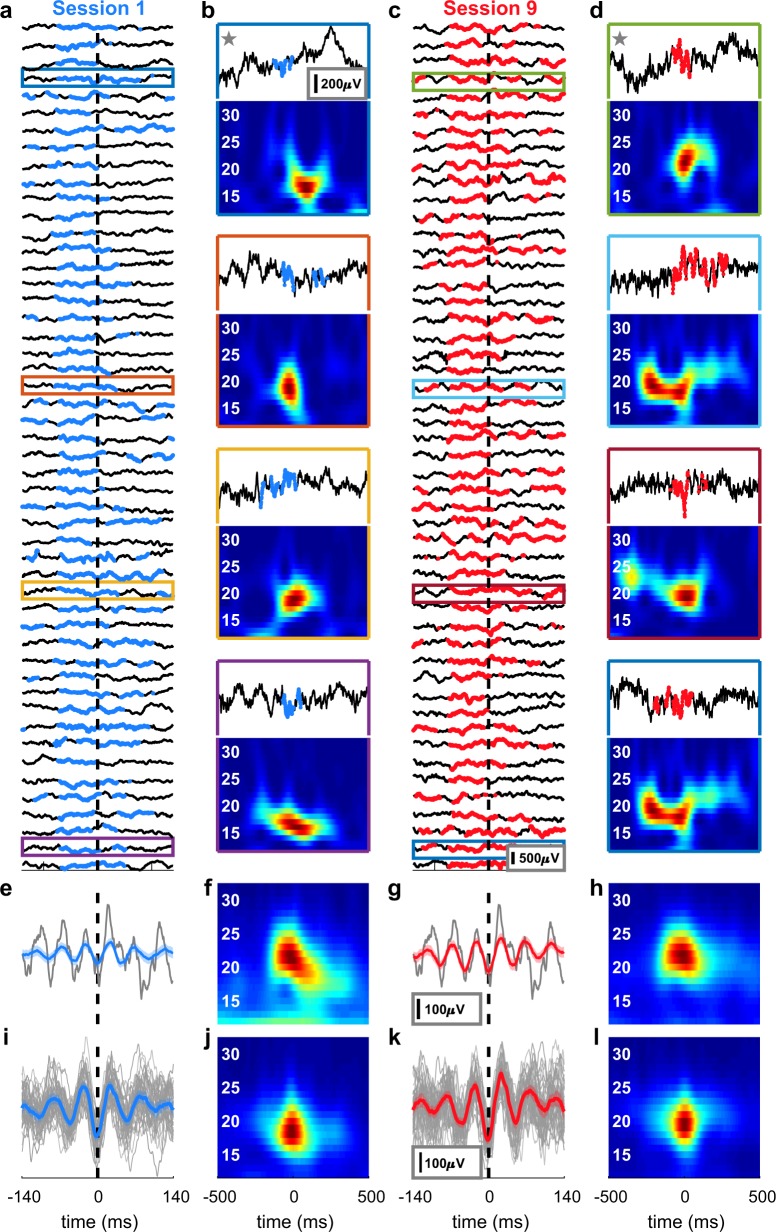
Examples and characterization of beta bursts. **a** Raw LFP traces of the first 50 rewards in the first session. **b** Examples of traces from **a** with the corresponding time-frequency representation. Coloured outlines refer to the traces in **a**. **c**, **d** Identical analysis as in **a** and **b** but for the last session. **e, g** Means of all bursts in the time domain for sessions 1 (blue) and 9 (red), respectively, aligned to the first trough of beta before a reward (black dashed line). Grey: raw trace of one example reward indicated with a star in **b** and **d** (respectively). **f, h** Means of all bursts in the frequency domain for session 1 and session 9, respectively. Although the bursts differed from each other in both the time and frequency domains, averaging led to a misleading appearance of a sustained oscillation (**e**, **g**) or a smoothed Gaussian (**f**, **h**). **i** Raw traces (grey) and mean (blue) of the strongest 50 bursts in the session exhibit a characteristic appearance of a trough and two smaller peaks, as previously described^5^. This standard waveform was preserved through training (**k**). **j, l** Time-frequency representation of **i** and **k** (respectively). Shaded areas in **e**, **g**, **j** and **k**: 95% confidence interval.

Within nine sessions of neurofeedback training per rat, oscillatory bursts became identifiable in raw LFP traces (Figs. 3a, b, 4a, c). Across the whole 30 min sessions, the averaged beta-power and the number of rewarded short-lived bursts were highly correlated (ρ = 0.89,  *p* = 5.9 × 10^−10^, Fig. 3c), despite the dynamic threshold (Supplementary Fig. 4). Individual bursts differed from each other in both the time and frequency domain (Fig. 4b, d), yet averaging all rewarded bursts of a session, aligned to the first trough before the reward, led to a misleading appearance of a sustained oscillation (Fig. 4e, g) or a smoothed Gaussian (Fig. 4f, h), as was reported recently^8^. Furthermore, the 50 strongest bursts (detected offline) were characterised by a trough and two smaller peaks surrounded by smaller oscillating nodes (Fig. 4i, k), or as a narrow Gaussian in the frequency domain (Fig. 4j, l), as described previously^5^. Notably, each rat had one prominent session with a sudden power increase (aha-effect, Fig. 3d, *p* < 1.55 × 10^−6^, Fig. 3g, *p* < 5.27 × 10^−6^, and Fig. 3j, *p* < 5.19 × 10^−6^, ANOVA with Bonferroni correction). This power increase occurred specifically in the targeted frequencies and only for rats receiving neurofeedback training (Fig. 3e, h, k, *p* < 0.01, *t*-test with Bonferroni correction), but could not be detected in the data of the sham-control rats (Figs. 3f and 2i). Analysis of the average beta-power per session of the neurofeedback-trained group revealed a significant increase in power in the last training sessions (sessions 7–9) compared to the average power of the initial three sessions (sessions 1–3, Fig. 3l, black traces, *p* < 0.00204, ANOVA). Importantly, the sham-control group did not show a similar power increase over sessions and significantly differed from the trained group (Fig. 3l, grey traces, *p* < 0.027, ANOVA). Taken together, these results indicate that rats increased the beta-bursts-related behaviour by neurofeedback training. In addition, these findings strongly support the critical influence of bursts on the global (i. e., averaged over a whole session) beta-band power, as was suggested previously^6^.

### β-bursts can be decoded from the movements of the rats

**Fig. 5:**
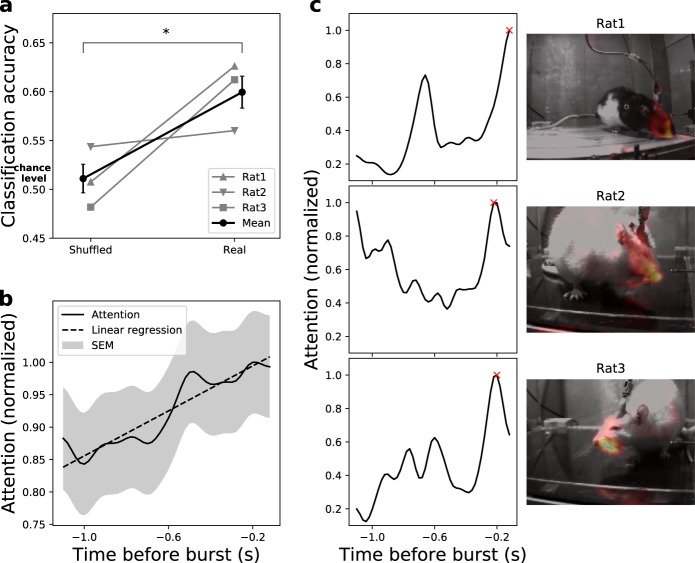
Behavioural effects of β-event neurofeedback training. **a** Classification accuracy of the SVM models (*n* = 3 repetitions) trained on epochs with correct labels vs. models trained on epochs with shuffled labels to infer chance levels. SVM models achieved above chance accuracy (18% increase, 0.6 vs. 0.51, two-tailed Welch *t*-test, t_(4)_ = 3.284 *p* = 0.03). **b** Temporal attention extracted from the SVM models on correctly predicted true samples. The time course of the mean normalized attention over all true positive epochs ± SEM indicates that movements correlated with beta bursts occurred primarily shortly before burst initiation (linear regression, ρ = 0.87, *p* = 2.8 × 10^−23^). **c** Examples of the model attention in individual epochs. The temporal (left panel) and spatial (right panel, including a heat map overlay for the video frame with highest attention, marked with a red x in the temporal profile) attentions of the model follow similar patterns for representative epochs of each rat. The attention implies that the movements of frontal body parts, shortly before burst initiation, were most informative to predict the epoch class.

In order to test for a link between the detected LFP bursts and behaviour, we analysed movements as behavioural readout, since we recorded from the motor cortex. Therefore, we performed video recordings of the behaviour of the rats synchronised with the LFP recordings. A critical matter for behavioural analysis is to avoid bias and maintain time-scale accuracy relevant to the underlying brain activity (tens to hundreds of milliseconds for LFP-bursts^1,8^). For a human observer, it is almost impossible to fulfil these criteria. Recently, it was suggested that an analysis based on machine learning can overcome these difficulties^14^. Therefore, we trained a support vector machine (SVM), a supervised classification algorithm, to predict the occurrence of neuronal LFP bursts from the videos in an offline manner (see Fig. 1c and Methods). We were able to link beta-bursts to behaviour, as the trained SVM model could reliably decode occurrences of bursts based on the rats’ movements with a prediction accuracy increased by 18% for true positive epochs compared to that of shuffled epochs (Welch *t*-test, *p* = 0.03, Fig. 5a). As input, the SVM received the optical flow between consecutive video frames. After the classifier was trained, the features (spatio-temporal subsets of the video frames) which were most relevant for predicting bursts were identified. We refer to those most informative features as the attention of the model. This unbiased attention increased during the trial towards burst initiation (ρ = 0.87, Fig. 5b), supporting the current view of increased power of beta oscillations at the termination of movements^15^. Additionally, the attention in space focused onto the frontal body parts of the rats (e.g. snout, Fig. 5c, right panel), indicating that indeed the rats’ movements were important for decoding LFP-bursts from the videos. Notably, despite the variability of freely moving behaviour, the movement-to-brain-activity classifier achieved classification accuracy comparable to brain-to-movement decoding of head-fixed animals^16^.

## Discussion

Here, we introduce a real-time LFP-burst-based neurofeedback system in freely moving rodents (Fig. 1). Previous animal studies have employed spike detection^17^, calcium transients^18,19^, and sustained LFP oscillations for neurofeedback^20,21^. Our results demonstrate for the first time the potency of real-time LFP transient burst detection for neurofeedback (Fig. 3). Furthermore, we confirm the impact of bursts on global oscillatory power and behaviour (Fig. 5), and characterize the overall distributions (Fig. 2) and averages (Fig. 4) of β-bursts in M1 in rats. The averaged LFP signal can be insightful, for example, for modelling standard shapes and the source of the signal^5^. However, comparing averaged signals across studies is problematic due to the impact of the chosen number of events, alignment points, and time spans on the averaged signal in both the time and frequency domains.

We focused on detecting and manipulating beta-bursts in the motor cortex, but the proposed algorithm is flexible and could be adjusted to target bursts in other frequency ranges and brain areas. Thus, our approach can be a starting point for a plethora of studies targeted at understanding the role of oscillatory bursts. The narrow-band targeting of individual frequencies allows investigating whether specific ranges of frequencies within the typically defined bands (alpha, beta, and gamma) are separable phenomena with different roles in behaviour. Further, instead of artificial external stimuli, real-time burst-triggered stimulus presentations could be combined with behavioural and electrophysiological measurements, thereby allowing to probe the intrinsic function of oscillatory bursts. Furthermore, neurofeedback has been used clinically for decades without a clear understanding of the underlying neural mechanisms^22^. As our tool is ideally suited for rodents, it can be combined with additional invasive or non-invasive treatments and post-mortem histology, thereby providing a new testbed with high relevance for future clinical developments, e.g. to advance the design and patient training of brain-machine interface prosthetic devices^22^.

## Methods

### Animals and surgery

In this study, we used adult female rats (*n* = 5, 56 ± 5 weeks of age, 347 ± 21 g, mean ± standard deviation at surgery day, four Sprague Dawley and one Long Evans, Charles-River, Sulzfeld, Germany, Supplementary Table 1), which were housed under an inversed 12 h light dark cycle. We implanted 32 IrOx electrode silicone probes (2 shafts, 150 μm between electrodes, model E32-150-S2-L6-200 NT, Atlas Neuroengineering, Leuven, Belgium) in the left motor cortices (2.4 mm lateral and 1.5 mm anterior to bregma). To anaesthetize the rats, we injected 80 mg/kg Ketamine (Medistar, Holzwickede, Germany) and 100 μg per kg Medetomidine (Orion Pharma, Espoo, Finland) intraperitoneally, as well as 10 mg per kg Carprofen (Rimadyl, Zoetis, Berlin, Germany) and 25 μg per kg Buprenorphine (Selectavet, Dr. Otto Fischer GmbH, Weyarn/Holzolling, Germany) as analgesics. To maintain vital body measures, a heating pad connected to a rectal temperature sensor (Stoelting, Dublin, Ireland) maintained the rat’s body temperature at 37 °C, and a pulse oximeter (model 2500 A VET, Nonin Medical, Plymouth, MN) monitored the blood oxygen level and heart-rate while delivering oxygen-enriched air (1 l per min) through a face mask. After placing the rat in the stereotactic frame (David Kopf Instruments, Tujunga, CA) and exposing and cleaning the skull, we thinned the bone above the motor cortex with a dental drill (MH-170, Foredom, Bethel, CT). A final small (~1 mm) craniotomy was made over a cortical area with no large blood vessels. We connected the flexible wire ribbon of the probe to an adaptor compatible with Tucker-Davis-Technologies (TDT, Alachua, FL) headstage’s zero-insertion-force (ZIF) connector, and held the ribbon on the stereotactic frame by a vacuum holder (Atlas Neuroengineering). As reference and ground, we connected 130 μm diameter silver wires (Science Products, Hofheim, Germany) and wrapped them around self-tapping screws (J.I. Morris Company, Southbridge, MA) positioned above the cerebellum. After lowering the probe until the tip reached 2 mm below dura, we applied a Kwik-Cast Sealant (World Precision Instruments, Sarasota, FL) over the craniotomy and a thin layer of super bond C&B cement (Sun Medical, Shiga, Japan) over the implant and supporting skull-screws. Afterwards, we added several layers of Paladur dental cement (Heraeus, Hanau, Germany) to cover the probe and adaptor, leaving only the ZIF connector of the adaptor exposed. To protect the connector, we attached a metal 780-11 paper-clip (ALCO, Arnsberg, Germany) to the adaptor. After the surgery, we placed the rat in a heated, oxygen-enriched chamber until it woke up, and administered Carprofen (10 mg per kg) and Buprenorphine (25 μg per kg) daily for 3 days. Rats were given >7 days to recover from surgery before water-restricted training began. All procedures were in accordance with the guideline RL 2010 63 EU and were approved by the Regierungspräsidium Freiburg.

### Real-time burst detection algorithm

Data acquisition and filtering: The real-time analysis of signals with amplitudes as small as a few microvolts demands for artefact-free recordings (see Supplementary Table 2 for artefact sources in electrophysiological recordings from freely moving animals and the measures taken to reduce their influence to a minimum) (Fig. 1b). We acquired raw broadband signals at 25 kHz using a digital headstage (ZD32, TDT) and downsampled them to 1 kHz. One electrode, located at a depth of 1100 μm (putatively layer 5), was selected for analysis. Filtering of the raw signal took place within the online digital signal processor (RZ2 BioAmp, TDT), using Bartlett window finite-impulse response (FIR) filters with a filter order of 256, a stopband attenuation of 6 dB and passband frequency width of 1 Hz. The filters were centred on frequencies ranging from 1 to 32 Hz in steps of 1 Hz. We generated the filter coefficients *b* with the Matlab (Mathworks, Natick, MA) function “fir1”, as follows: *b* = fir1(*N*, [*Fc1 Fc2*]/(*Fs*/2), ‘bandpass’, *win*, *flag*) with the following parameters: filter order *N* = 256; central frequency *Fc*  from 1 to 32 Hz in steps of 1 Hz; lower limit of passband *Fc1* = *Fc*−0.5 Hz; upper limit of passband *Fc2* = *Fc* + 0.5 Hz; sampling frequency *Fs* = 976.5625 Hz (~1 kHz as implemented in the TDT system); window *win*: Bartlett window of order 257 (*N* + 1) generated by the Matlab function “bartlett”; normalizing the magnitude response of the filter at the centre of the passband is performed by setting *flag* = 1. With these parameters, the filter has a full width at half magnitude (FWHM) of 5 Hz (Supplementary Fig. 1) and a sample delay of 128 (*N*/2) ms. The total delay of the filter (group delay + computation time of 2 ms) was 130 ms for each frequency, allowing direct comparisons between frequencies.

Two video cameras (Basler acA640-750um) recorded the rats’ movements from orthogonal viewpoints. To ensure that video frames were synchronized with the electrophysiological data, the acquisition system triggered the cameras via a transistor–transistor logic (TTL) signal (50 Hz square wave with 40 μs width).

Power and phase estimation: The real-time algorithm detected turning points in each frequency by applying a peak/trough feature detection routine on the frequency-filtered signal. The squares of the LFP amplitudes at the detected points served as power estimates. Latching a power value until the following extrema point detection yielded a time resolution of half the period of each frequency. Although phase estimation was not used in the current study, the algorithm estimated the phase by syncing a saw-tooth signal with amplitude 2π and frequency f to 0 upon detection of a peak point and to π upon detection of a trough. To align the determined phase to the true phase, the phase-reset was delayed by δ(*f*) = ⌈*d/T*⌉*T-d, where δ(*f*) is the delay in seconds to the next peak, *f* is the frequency in Hz, *d* is the group delay of the filter in seconds, *T* the time period in seconds and ⌈ ⌉ the ceiling operator. This delay compensated for the filter group delay, and aligned the phase to the next assumed peak of the real signal.

Online artefact rejection: Rare events of high amplitude LFP deflections (which occurred mainly when the rats crunched their teeth or touched the headstage during grooming) have extremely high power in a large range of frequencies. These artefacts might be erroneously interpreted as bursts, and have a tremendous effect on the power distribution. Therefore, LFP values from the selected electrode, which exceeded a threshold of 500 μV, were considered artefacts, and data points 500 ms before and after the detected artefact were removed from analysis. When an artefact was detected, the rat received 1 s of 90 dB SPL white noise and a no-reward timeout, to train it to avoid causing artefacts. To avoid the false positive detection of artefacts by high frequency action potentials or very low frequency drifts of the sensors, the raw LFP went through a 12 dB per octave Butterworth bandpass filter between 2 and 250 Hz prior to thresholding. Overall, 0.57% ± 0.1 (mean ± SEM) of samples per session were rejected.

Burst detection: The real-time DSP buffered the power in each frequency together with detected artefact times and sent them to a Matlab routine for the dynamic estimation of the percentiles every second. The Matlab routine used the last 15 acquired seconds for calculation, while ignoring LFP-power values marked as artefacts. To substitute missing values during artefact rejection times, the routine used earlier values, assuring full 15 s of artefact-free data for percentile calculation. The percentile-based power threshold of each frequency was sent back to the DSP for real-time burst detection; in every time point, the DSP compared the power value in each frequency to the target power threshold as well as to the power of the neighbouring frequencies. If the power in a target frequency range (15–20 Hz for 1 rat, 20–25 Hz for 2 rats) exceeded these values, the DSP sent a TTL pulse to the behavioural controller (Med Associates, Fairfax, VT), signalling a burst.

### Neurofeedback training

To obtain clear video images and to avoid artefacts caused by electrostatic discharges, we built an open-top glass cage sized 30 × 26 × 40 cm (width × length × height) and positioned it inside a grounded Faraday cage. A 2 × 12 mm infusion cannula (1464LL, Acufirm, Dreieich, Germany) served as a spout for 3% sucrose water delivery as reward for the water deprived rats, and was controlled by an infusion syringe pump (PHM-107, Med Associates, see Fig. 1a). In order to allow time for the initial period of percentile computation, in the first 15 s of the session, the rats received 5 rewards of 50 μl sucrose water delivered every 3 s. Henceforth, each session lasted 30 min. Upon detection of a rewarded burst from the DSP lasting longer than 70 ms, the rats obtained 30–75 μl sucrose water rewards delivered at 50 μl/second. The reward size was adjusted to ensure that the rat received 8–14 ml water per day, and was accompanied by a 12 kHz, 90 dB SPL pure tone to facilitate learning. During reward delivery and 1 s after a reward or an artefact, no reward could be obtained. While all surgical and recording procedures for the rats in the sham-control group (*n* = 2) were identical to the neurofeedback-trained group (*n* = 3), they received sucrose water and tones at time points during which the trained rats 1 and 2 (respectively) had been rewarded, regardless of their current brain-activity. Essentially, we replayed the reward history of the trained rats to the sham-controls. Training lasted nine sessions (1–2 sessions per day) during the dark period. We weighed the rats before each session to assure they stayed above 80% of their pre-deprivation weight.

### Offline machine learning and video analysis

Flow calculation: To relate the occurrence of beta bursts to behaviour, we analysed the apparent movements of the rats. To extract movement-related features from the videos, we used optical flow (Fig. 1c) using FlowNet 2.0^23^ (https://github.com/lmb-freiburg/flownet2), which estimates the pixel changes between two images, resulting in an *x*- (*u*) and *y*- (*v*) vector for every pixel between two consecutive images. Individual frames from one of the cameras were extracted via ffmpeg (2.8.15^24^), scaled down to 320 × 240 pixels and passed through FlowNet 2.0 to calculate the optical flow between the frames.

Data preparation: Time points of beta bursts as detected online were used during the offline analysis to extract the corresponding frames. We used epochs of 50 frames (corresponding to 1 s) from 1.1 to 0.1 s before the time of the beta-burst as input to the classifier. Time points during reward delivery were excluded from the analysis to avoid the detection of the reward itself by the model. Negative samples (i.e. periods with no detected beta bursts) were randomly chosen time points of identical length (i.e. 50 frames), which did not overlap with the rewarded epochs. The ratio between positive and negative samples was kept at 1:1 for each session. The data were randomly separated (while keeping the ratio between positive and negative samples) into training and test sets for each training run.

Support vector machine (SVM) classifier: To test for differences in behaviour during beta burst epochs as compared to epochs without beta bursts, we employed a supervised linear classifier. To handle the large flow files (60 GB per 30 min session), we used an out-of-core incremental implementation of the support vector machines algorithm (sklearn^25^ 0.20.3).

The input data (samples × time points × frame width × frame length × motion dimensions) were z-scored (mean subtracted and divided by the standard deviation, calculated pixel-wise on the training set) and reshaped into a 1D array. Our SVM model was implemented as stochastic gradient descent classifier (SGDClassifier^26^) with hinge loss and L2 regularization (alpha = 0.0001). We evaluated the model accuracy on hold-out sets as a mean of 3-fold splits of the data. Models receiving the same features as inputs but shuffled labels served as controls. No hyperparameter optimisation was performed.

Model attention: Using a linear algorithm for classification allowed for projecting the model decision function back onto inputs and, thereby, for analysing the input subspace (i.e. frame pixels) leading to the correct prediction. Model attention was defined as the distance of the input data point to the decision function. SVM fits a hyperplane (decision function) to the training data, which separates the classes with highest margin. Thus, for each data point, we calculated on which side of, and with which distance to the hyperplane the data point was located. Afterwards, we determined the time and space of the most important points, i.e. the points contributing the most to the correct classification. For representation purposes, attention was filtered with a [0,2,3,3,0] Gaussian. Temporal attention was calculated as a sum of values over *x, y, u*, and *v* dimensions per epoch and normalized to its maximum. Spatial attention was analysed for time points with maximal temporal attention within an epoch.

### Offline power and behavioural analysis

Characterization of beta bursts: to evaluate the distribution of the entire burst population in a session, the time-frequency-representation (TFR) was obtained by Morlet wavelets with a width of 7 cycles and steps of 1 ms using the fieldtrip toolbox^27^. Bursts were defined as peaks in the time-frequency plane^8^ exceeding the 98th power percentile^5^. While the peak frequency (Fig. 2a) could directly be derived, the frequency span (Fig. 2b) was defined as the distance (in Hz) between the lowest and highest frequencies with a power above the 98th percentile at the peak time point. The burst duration (Fig. 2c) was defined as the distance (in ms) between the first and last time point with a power above the 98th percentile of the peak frequency. The difference (in time) between subsequent peaks was defined as the inter-burst-interval (Fig. 2d). The power in Fig. 2e is presented as the spectrogram value at the peak time and frequency, normalized to the median of that frequency over the whole session.

The spectrograms in Supplementary Figs. 1 and 2 represent the power as estimated by an array of filters to simulate the real-time algorithm. The power in Fig. 4 was obtained by Morlet wavelets with a width of seven cycles. In all spectrograms, the power was normalized by the ratio of the 98th percentile of each frequency. The mean session time-locked averages were calculated by aligning the raw LFP of each rewarded burst (Fig. 4e, g) or of the strongest 50 bursts (Fig. 4j, l) to the last trough of beta before reward initiation, and then averaging over the session. This trough was detected offline by bandpass filtering the raw trace at 15–30 Hz, then using the Matlab’s peak finding routine (“findpeaks”) on the negative of the filtered data. To demonstrate the flexibility of the proposed method, we targeted two different ranges of beta oscillations: low-beta (15–20 Hz) in one rat and medium-high beta (20–25 Hz) in two rats (Supplementary Table 1). In order to investigate group effects, and due to the 1/f nature of LFP signals^28^, we calculated the normalized beta-power (Fig. 3d, g, j, l) as follows; the power of each frequency and session (as computed online) was averaged over the entire session (ignoring epochs of rejected artefacts). The session mean was then normalized to the mean of the first session, and the change of individual targeted beta frequencies was averaged and translated into percentages:

1 $${\mathrm{Beta}}\,{\mathrm{power}}\,{\mathrm{change}}\left( s \right) = \frac{{\mathop {\sum }\nolimits_{f = F_1}^{F_n} \left( {\frac{{\mathop {\sum }\nolimits_{t = 1}^{T_s} P_s\left( {f,t} \right)}}{{T_s}}/\frac{{\mathop {\sum }\nolimits_{t = 1}^{T_1} P_1\left( {f,t} \right)}}{{T_1}}} \right)}}{n}\ast 100 - 100$$

with *s* denoting the session, *F* = targeted frequencies, *f* = individual frequencies, *n* = 6 targeted frequencies, *Ts* = total number of time points s in a session, *t* = individual time points (samples) and *Ps* = power of session s. This value was averaged again over all rats to yield the group beta-power change.

Within-subject analysis: to investigate the effect of training on a wide range of frequencies (5–100 Hz) in individual rats (Fig. 3e, f, h, I, k), we compared the mean TFR 200 ms before a reward (the beginning of the burst, with minimal length of 70 ms and 130 ms delay) to the mean TFR 200 ms after the reward. This value was contrasted between the first and last session, using a *t*-test with Bonferroni correction, as follows:

2 $${\mathrm{Training}}\,{\mathrm{effect}}\,\left( f \right) = \left( {\bar P_{9,\,{\mathrm{pre}}} - \bar P_{9,\,{\mathrm{post}}}} \right) - \left( {\bar P_{1,\,{\mathrm{pre}}} - \bar P_{1,\,{\mathrm{post}}}} \right)$$

with $\bar P$ denoting the mean power (digit in subscript denoting the session number), pre 200 ms before reward and post 200 ms after reward.

Comparison to other methods: We compared the proposed online filter-extrema method to three other conventional time-frequency decomposition methods: wavelet analysis, fast Fourier transform (FFT) and variance of filtered data (variation). For each method, two sets of parameters were used, denoted  “offline” as commonly used in offline analyses and “online” in which we applied computation time constraints similar to those of our online methods (delay of 130 ms + half the period of each frequency). For each of the analyses, we divided the raw data into 100 “trials” (reward timestamps ± 0.5 s) and padded with 1 s before and after the trial. The wavelet and FFT decompositions were computed using the fieldtrip toolbox^22^. For wavelet analyses, we used Morlet wavelets with a width of seven cycles (offline) or three cycles (online, corresponding to 130 ms + half the time period in 20 Hz), in steps of 1 ms. For FFT, we used Hanning time windows of 250 ms (offline) or 150 ms (online), in steps of 1 ms. The variation was computed by filtering the data (Matlab function “bandpass”, a Kaiser window FIR filter with stopband attenuation of 12 dB, passband of 1 Hz, centred at the frequencies of interest and with a steepness of 0.75). Afterwards, the variance in each frequency was calculated over a window of 150 ms (offline) or half the time period of each frequency (online), sliding in 1 ms steps.

To compute the sum square error (*SSE*) metric, we normalized the power estimation of each method by the median of all trials. We applied the commonly used seven-cycle wavelet as the gold standard, and subtracted the 2D matrix (time x frequency) of each trial from the wavelet values of this trial. The difference was squared and summed over time and frequency, as follows:

3 $${\mathrm{SSE}}\left( {m,n} \right) = \sum_{t = 1}^{1000} \sum_{f = 12}^{32} {\left( {\frac{{P\left( {m,n,t,f} \right)}}{{P_{50}\left( m \right)}} - \frac{{P\left( {w,n,t,f} \right)}}{{P_{50}\left( w \right)}}} \right)^2}$$

with *m* denoting the method, *n* trial number (1–100), *t* time point (ms), *f* frequency (Hz), *w* wavelet with sevencycles width, *P* power and *P*_*50*_ median power.

Simulations: in order to test the performance of the filter-envelope method, we required ground truth data with respect to the occurrence of bursts. To this end, we generated a set of surrogate data, composed of a pair of bursts with a known amplitude (set to 1), and adjacent frequencies (20 and 21 Hz), by multiplying a sinusoid and a Gaussian. To these artificial bursts we added pink noise, generated by filtering white noise with a 1/f passband filter^29^ with amplitude = 1.5 and white (random) noise with amplitude = 0.3 (Supplementary Fig. 1b). This surrogate LFP trace was injected to a filter-envelope algorithm executed in Matlab, which returned the frequencies and time points (samples) of detected bursts. To test whether the system is capable to detect bursts in 1 Hz resolution, we repeated this procedure 50 times. As a statistic measure, we used the z-value of the Wilcoxon rank sum comparison of the first versus the second peak in all simulations. For bootstrapping, we calculated the z-value of 10,000 random assignments of detected frequencies to the first or second burst (keeping the number of peaks identical). The p-value reported in Supplementary Fig. 1d represents the probability of a random assignment to yield a higher z-value than the real simulated data.

### Statistics and reproducibility

Statistical tests were performed in Matlab. To compare the SSE means of the different decomposition methods over trials (Supplementary Fig. 3e), we used one-way analysis of variation (ANOVA). Two-way ANOVA was applied in Fig. 3d, g, j, l. Whenever conducting multiple comparisons in post-hoc analyses, we used Bonferroni correction. To test linear regression, we computed Pearson’s correlations. The minimal number of animals needed for the study was determined using the resource equation approach^30^: Minimum *n* = ⌈10/(s–1) + 1⌉, with *n* = 3, number of rats and s = 9, number of sessions.

### Reporting summary

Further information on research design is available in the Nature Research Reporting Summary linked to this article.

## Supplementary information

Supplemental Information

Supplementary Movie 1

Supplementary Data 1

Supplementary Data 2

Supplementary Data 3

Supplementary Data 4

Description of Additional Supplementary Files

Reporting Summary

## Data Availability

The datasets generated and/or analysed during the current study are available in the GIN repository, [https://gin.g-node.org/optophysiology/Neurofeedback]. All source data underlying the graphs and charts presented in the main figures are available as supplementary information files (supplementary data 1–4).

## Code availability

Code for acquisition and analysis of the neurofeedback experiments is available on Github (https://github.com/Optophys/Neurofeedback, 10.5281/zenodo.3600375)^31^. The data and code are shared under the Attribution ShareAlike (CC BY-SA) license.
