# Supplementary material for: Real-time detection of neural oscillation bursts allows behaviourally relevant neurofeedback
Source: Commun Biol. 2020 Feb 14;3:72. doi: 10.1038/s42003-020-0801-z (PMC7021904; doi:10.1038/s42003-020-0801-z)
Supplement: Supplementary file 1 — Supplemental Information [file 42003_2020_801_MOESM1_ESM.pdf]

Real-time detection of neural oscillation bursts allows behaviourally relevant neurofeedback

Supplementary material

Supplementary figures:

- Supplementary figure 1: The filter envelope method allows detection of bursts with 1 Hz resolution (Related to figure 1).
- Supplementary figure 2: Supplementary Figure 2- Optimizing the frequency-time resolution trade-off (Related to figure 1).
- Supplementary figure 3: The filter-envelope method outperforms conventional methods in online beta-burst detection (related to figure 1).
- Supplementary figure 3: Power threshold dynamics (related to figure 3).

Supplementary tables:

- Supplementary Table 1: details of the rats used in the study.
- Supplementary Table 2: Sources of artefacts in electrophysiological recordings from freely moving rodents, and the measures to reduce their influence.

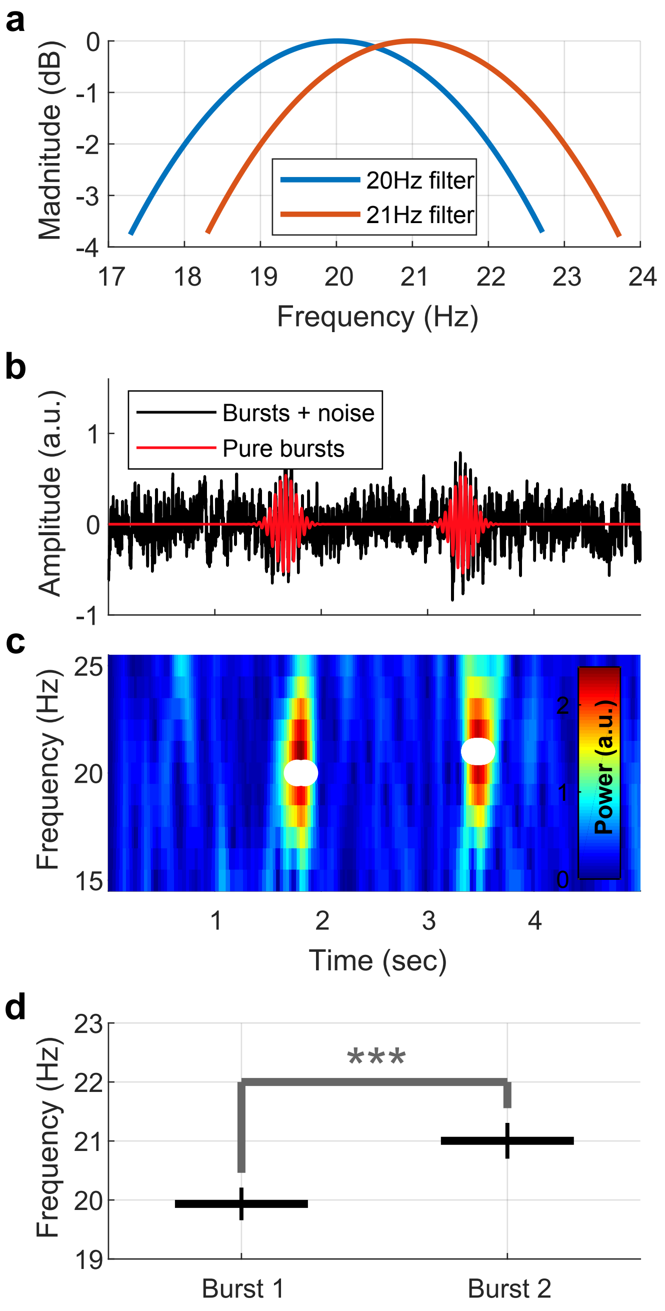

Supplementary Figure 1- The filter envelope method allows detection of bursts with 1 Hz resolution (Related to figure 1).

a. frequency-amplitude characteristics of two of the digital filters (20 and 21 Hz). The full width at half maximum (i.e. -3 dB) is 5 Hz. b. Simulation of 2 bursts, the first peaked at 20 Hz and the second peaked at 21 Hz, with added 1/f (pink) noise, with amplitude 1.5 times bigger of the maximal peak power, and white noise with 0.3 of the peak power. c. Time-frequency representation of the simulated data in b based on the filter-envelope method. White dots: detected peaks. Despite the filter bandwidth of 5 Hz, the burst-detection resolution is 1 Hz. d. Mean ± SD of the peak frequencies detected at the time points of the simulated bursts (50 simulation repetitions). ***- $P=10^{-4}$, bootstrap.

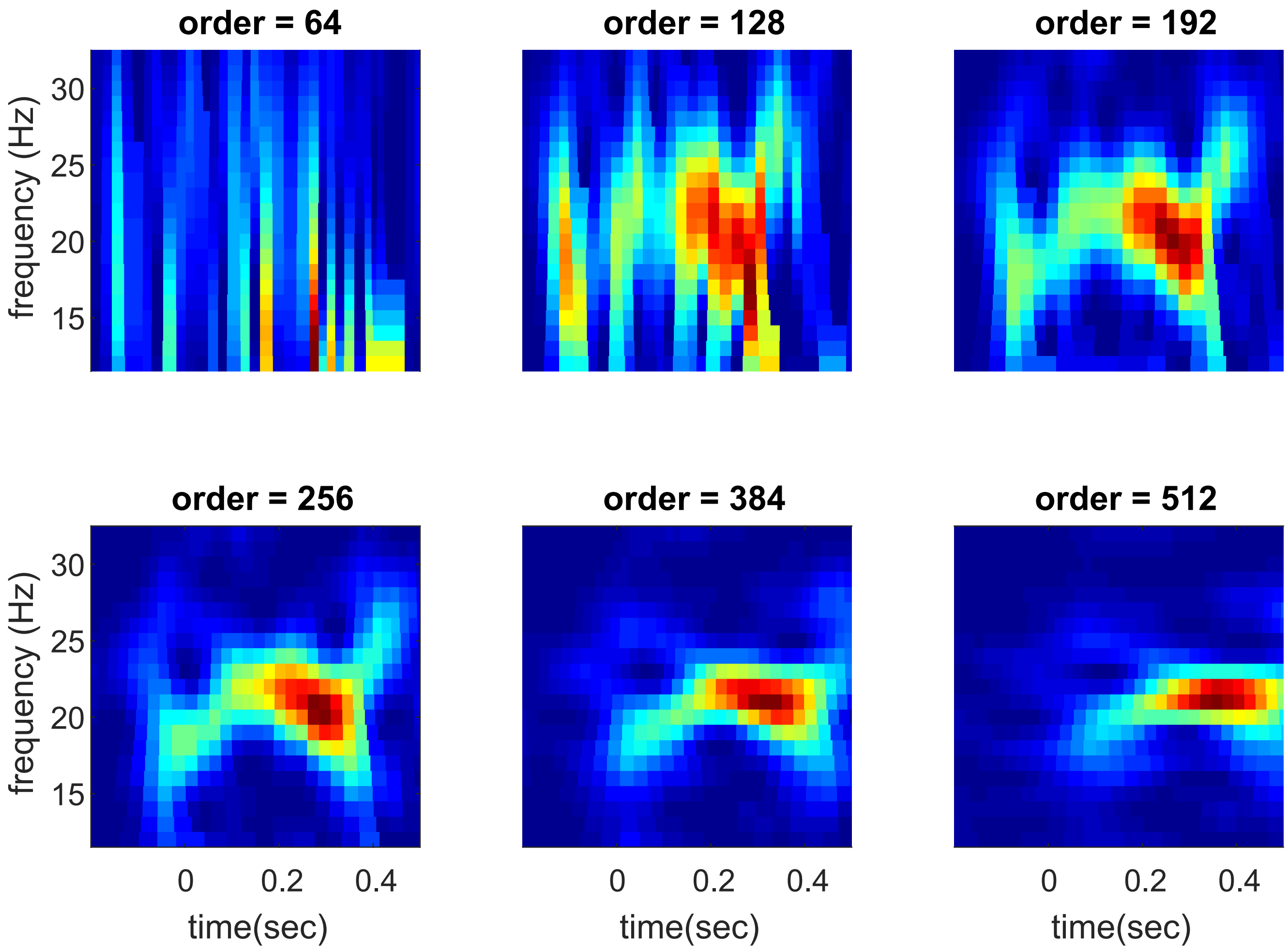

Supplementary Figure 2- Optimizing the frequency-time resolution trade-off (Related to figure 1).
Time-Frequency representation of one beta-burst using Bartlett FIR filters with different size orders. Filters with bigger orders have a higher quality factor (narrowness of the filter) and respond slower. Note that filters with orders smaller than 192 lack the frequency resolution to detect a burst, and filters with orders higher than 256 do not contribute essential information regarding the peak frequency. In this study, we used a filter order of 256.

Supplementary figure 3

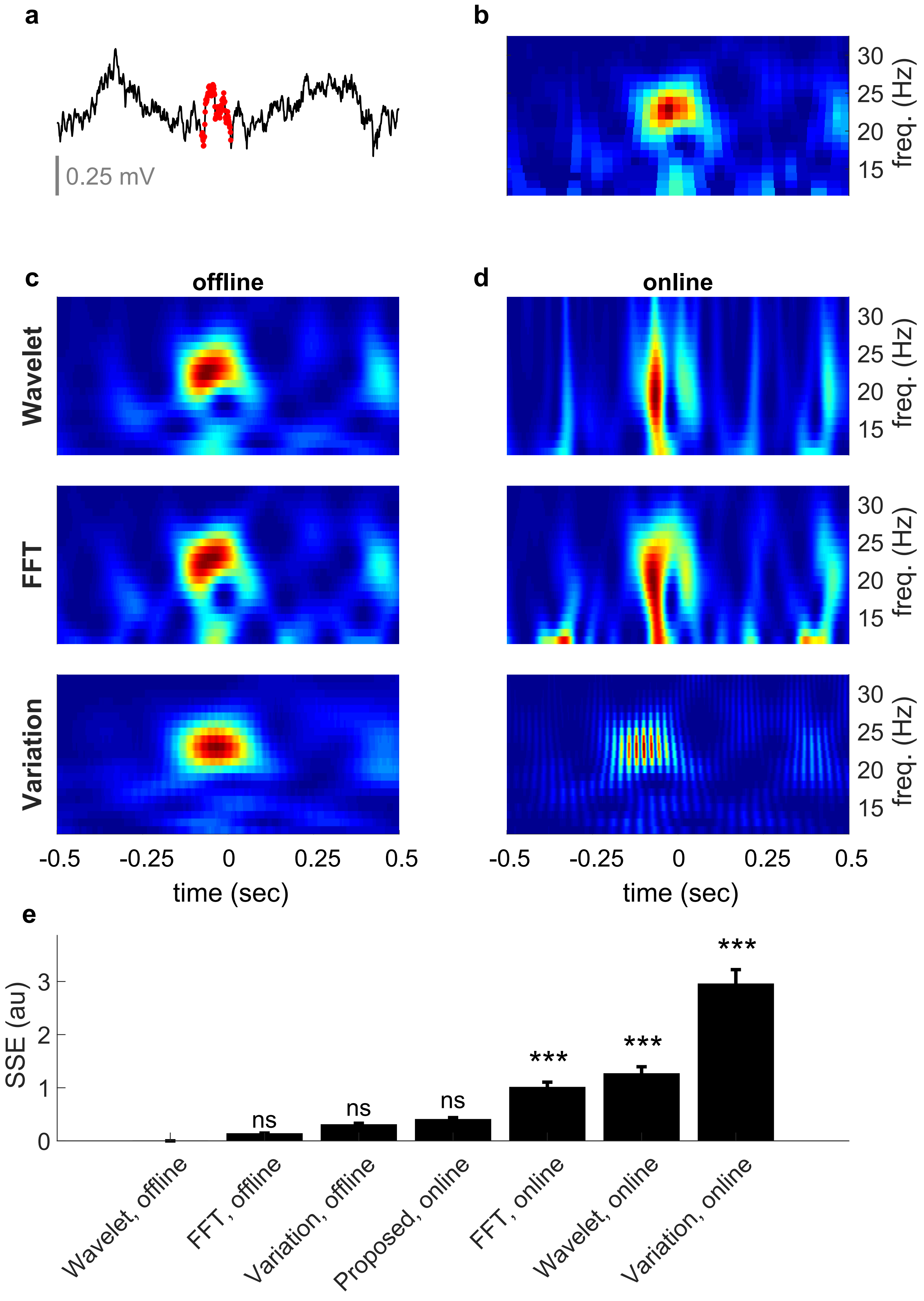

Supplementary Figure 3: The filter-envelope method outperforms conventional methods in online beta-burst detection (related to figure 1).

a. An LFP trace of a rewarded beta-burst ± 0.5 sec. Time-points in which beta-power exceeded the 98th percentile threshold are marked in red. Reward was delivered at time = 0. **b**. Time-frequency decomposition of the trace in **a** using our online filter-extrema method. **c**. Time-frequency decomposition of the trace in A using three conventional offline analysis methods: convolution with Morlet wavelets with width of 7 periods ("Wavelet", top), fast Fourier transform with sliding Hanning window sized 250 ms ("FFT", middle) and the variance of the filtered data computed over windows of 150 ms ("Variation", bottom). **d**. Applying the same time constraints as in the online filter-extrema method (delay of 130ms + half the period of each frequency) in the methods in **c**, resulted in worse resolution in the frequency dimension (Wavelet, top, and FFT, middle) or distortion in the time dimension (Variation, bottom). Wavelet and FFT were used with 3 periods and 150 ms accordingly to match the time delay for 20Hz. Variation was calculated over half the time period of each frequency. Note that for the wavelet, FFT and filter-extrema methods it is possible to extract the phase, while for the variation method, phase cannot be determined. **e**. The commonly used 7 periods wavelet was used as a standard, to which each method was compared to compute the sum of the square of the error (SSE) in 100 epochs of rewarded beta-bursts ± 0.5 sec. The proposed filter-extrema online method did not differ significantly from the offline methods, while all other online methods did (one-way ANOVA, $F(6,693) = 74.006$, $p=2.87*10^{-71}$). Methods are sorted according to similarity to the offline wavelet method, and presented as mean ± SEM. ns- no significant difference in comparison to offline wavelet. ***- $p<10^{-7}$, after Bonferroni correction for multiple comparisons.

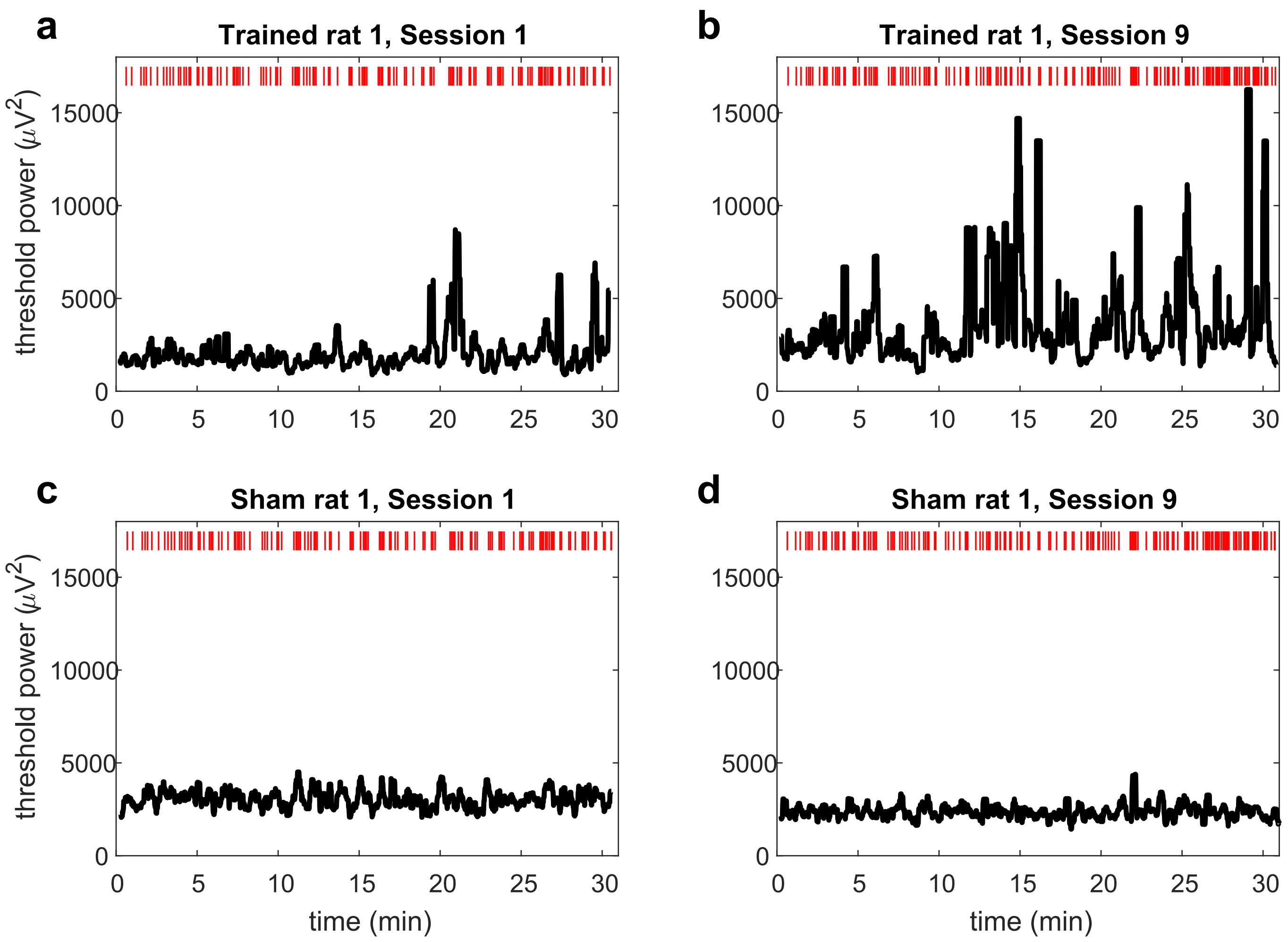

Supplementary Figure 4- Power threshold dynamics (related to figure 3).
The 98th percentile of the LFP power at 20 Hz is plotted as a function of time, for the first (a and c) and last (b and d) session of neurofeedback (a and b) or sham (c and d) training. Red lines represent water delivery times. The percentile was calculated for every second over the preceding 15 seconds, and served as the power-threshold in the real-time burst detection algorithm.

Supplementary Table 1: details of the rats used in the study

**Group**	**Trained**			**Sham**	
**Rat number**	**1**	**2**	**3**	**1**	**2**
Rat designation number	206	371	373	480	481
Strain	Long Evans	Sprague Dawley	Sprague Dawley	Sprague Dawley	Sprague Dawley
Age at surgery (weeks)	49	60	59	36	36
Age at training initiation (weeks)	94	65	65	40	40
Weight at surgery (g.)	370	360	322	325	360
Weight at end of training (g.)	380	350	310	305	340
Session of aha-moment	4	7	6	-	-
Target frequencies (Hz)	20-25	15-20	20-25	-	-

Supplementary Table 2: Sources of artefacts in electrophysiological recordings from freely moving rodents, and the measures to reduce their influence*.

**Artefact source**	**Artefact reduction measures**
Alternating electric fields from the network supply (50/60 Hz hum)	Position the experimental setup in a grounded Faraday cage.<br>Uncouple the animal and recording pathway from the ground of the building (e.g. by optical interfaces).
Capacitance changes in moving electrical cables/ connections (microphony)	Digitize and amplify the signal at the headstage level.<br>Cement adaptors to avoid movement of connectors.<br>Keep the cable path clear of obstacles and tangling by an open-top cage and a commutator.<br>Reduce strain form the cable (in the "z" axis) by attaching soft springs to it.<br>Ensure clean and tight implant, with multiple reference points.
Electro-static discharges	To build the cage, use materials triboelectrically similar to the animal's fur (e.g., glass). Avoid poly (methyl methacrylate), also known as Plexiglass or Perspex.<br>Wear a grounding wrist strap when handling the rats.
Alternating magnetic fields	Disconnect electrical transformators from sockets.
Switching devices (usually from the behavioural apparatus, e.g. lights and reward ports)	Keep high voltage devices out of the Faraday cage.<br>Use shielded and grounded cables.
Muscular activity, especially face muscles	To reduce epochs of teeth crunching, maintain the subject motivated and satisfied (e.g. by adjusting the reward size).<br>Detect epochs of facial muscles activity/ touching the headstage and remove them from analysis.
Direct-current discharges from touching the ground	Keep the rat and recording system floating above ground (by isolating materials e.g. glass and wood).<br>Keep large conductive materials out of the cage.

* see also: https://neuralynx.com/news/techtips
