# Supplementary material for: Real-time detection of neural oscillation bursts allows behaviourally relevant neurofeedback
Source: Commun Biol. 2020 Feb 14;3:72. doi: 10.1038/s42003-020-0801-z (PMC7021904; doi:10.1038/s42003-020-0801-z)
Supplement: Supplementary file 7 — Description of Additional Supplementary Files [file 42003_2020_801_MOESM7_ESM.docx]

**Supplementary data 1: source data for figure 2.**

Microsoft® Excel® file, with the maxima frequency (Hz), frequency span (Hz), duration (ms) power (mV^2), power (ratio to 98^th^ percentile and to median) of all bursts (detected offline) used for the distribution analysis if figure 2. Sessions 1-3 are represented in the figure as blue bars, and sessions 7-9 as a red line.

**Supplementary data 2: source data for figure 3.**

Microsoft® Excel® file, with the data underlying each panel shown in an individual spreadsheet.

**Supplementary data 3: source data for figure 4.**

Microsoft® Excel® file, with the data underlying each panel shown in an individual spreadsheet. Note that the data used for panels b and d is given in panels a and c, respectively.

**Supplementary data 4: source data for figure 5.**

Microsoft® Excel® file, with the data underlying each panel shown in an individual spreadsheet.

**Supplementary Movie 1: Video of a freely moving rat with raw LFP trace and power estimation (related to figure 1)**

Example video of the freely moving rat (left) with a raw LFP trace (bottom right) and the power estimation as computed online (spectrogram, top right). The colour map is normalized to the 98^th^ percentile of the power in each frequency, i.e., values higher than 1 are above the statistically defined threshold. If the power in a specific frequency crossed the threshold and was also higher than the neighbouring frequencies, it was considered a burst and denoted with a white overlay (over the spectrogram) or red overlay (over the LFP trace). If a burst lasted >70 ms, the rat was rewarded with sucrose water (blue lines above the LFP trace). The next burst could be rewarded only after the end of reward delivery. Note that the delay between the LFP trace and the power estimation is constant at 130 ms, which is due to the group delay of the online filters.
