# Supplementary material for: Real-time detection of neural oscillation bursts allows behaviourally relevant neurofeedback
Source: Commun Biol. 2020 Feb 14;3:72. doi: 10.1038/s42003-020-0801-z (PMC7021904; doi:10.1038/s42003-020-0801-z)
Supplement: Supplementary file 8 — Reporting Summary [file 42003_2020_801_MOESM8_ESM.pdf]

nature research

Corresponding author(s): Ilka Diester

Last updated by author(s): Dec 24, 2019

Reporting Summary

Nature Research wishes to improve the reproducibility of the work that we publish. This form provides structure for consistency and transparency in reporting. For further information on Nature Research policies, see Authors & Referees and the Editorial Policy Checklist.

Statistics

For all statistical analyses, confirm that the following items are present in the figure legend, table legend, main text, or Methods section.

n/a	Confirmed	
☐	☒	The exact sample size (*n*) for each experimental group/condition, given as a discrete number and unit of measurement
☐	☒	A statement on whether measurements were taken from distinct samples or whether the same sample was measured repeatedly
☐	☒	The statistical test(s) used AND whether they are one- or two-sided<br>*Only common tests should be described solely by name; describe more complex techniques in the Methods section.*
☐	☒	A description of all covariates tested
☐	☒	A description of any assumptions or corrections, such as tests of normality and adjustment for multiple comparisons
☐	☒	A full description of the statistical parameters including central tendency (e.g. means) or other basic estimates (e.g. regression coefficient) AND variation (e.g. standard deviation) or associated estimates of uncertainty (e.g. confidence intervals)
☐	☒	For null hypothesis testing, the test statistic (e.g. *F*, *t*, *r*) with confidence intervals, effect sizes, degrees of freedom and *P* value noted<br>*Give P values as exact values whenever suitable.*
☒	☐	For Bayesian analysis, information on the choice of priors and Markov chain Monte Carlo settings
☒	☐	For hierarchical and complex designs, identification of the appropriate level for tests and full reporting of outcomes
☐	☒	Estimates of effect sizes (e.g. Cohen's *d*, Pearson's *r*), indicating how they were calculated

Our web collection on statistics for biologists contains articles on many of the points above.

Software and code

Policy information about availability of computer code

Data collection	The circuit for real-time analysis and data collection was realized in Real-time Processor Visual Design Studio (RPvdsEx), from Tucker Davis Technologies and is available in Github
Data analysis	Offline analysis of electrophysiological and behavioral data was performed in Matlab R2018a . Machine learning analysis was performed in python and the code is available in Github

For manuscripts utilizing custom algorithms or software that are central to the research but not yet described in published literature, software must be made available to editors/reviewers. We strongly encourage code deposition in a community repository (e.g. GitHub). See the Nature Research guidelines for submitting code & software for further information.

Data

Policy information about availability of data

All manuscripts must include a data availability statement. This statement should provide the following information, where applicable:

- Accession codes, unique identifiers, or web links for publicly available datasets
- A list of figures that have associated raw data
- A description of any restrictions on data availability

Code and example data sets are available on Github (https://github.com/arturoptophys).

Field-specific reporting

Please select the one below that is the best fit for your research. If you are not sure, read the appropriate sections before making your selection.

☒ Life sciences ☐ Behavioural & social sciences ☐ Ecological, evolutionary & environmental sciences

Life sciences study design

All studies must disclose on these points even when the disclosure is negative.

Sample size	This study was designed with an exploratory nature, hence it was not possible to estimate effect size and variation a-prioi, which are necessary for power analysis. As an alternative, we used the 'resource equation' approach, stating that the acceptable range of degrees of freedom for the error term in an analysis of variance (ANOVA) is between 10 to 20 (Festing & Altman, doi: 10.1093/ilar.43.4.244; Arifin & Zahiruddin, doi: 10.21315/mjms2017.24.5.11). For repeated measures within subjects, the minimal number of animals can be determined by the formula: n = ceil (10/(s–1) + 1). For 9 sessions, the minimal n is 3.
Data exclusions	No data was excluded
Replication	Replication of the LFP recording findings relied upon the collection of neuronal data from independent animals. The machine learning findings were based on three replications for each rat.
Randomization	The first version of the manuscript included only the neurofeedback-trained group. The sham-treated group was added later.
Blinding	Blinding was not relevant as the behavioral analysis was decoded by an unbiased machine learning algorithm.

Reporting for specific materials, systems and methods

We require information from authors about some types of materials, experimental systems and methods used in many studies. Here, indicate whether each material, system or method listed is relevant to your study. If you are not sure if a list item applies to your research, read the appropriate section before selecting a response.

Materials & experimental systems

n/a	Involved in the study	
☒	☐	Antibodies
☒	☐	Eukaryotic cell lines
☒	☐	Palaeontology
☐	☒	Animals and other organisms
☒	☐	Human research participants
☒	☐	Clinical data

Methods

n/a	Involved in the study	
☒	☐	ChIP-seq
☒	☐	Flow cytometry
☒	☐	MRI-based neuroimaging

Animals and other organisms

Policy information about studies involving animals; ARRIVE guidelines recommended for reporting animal research

Laboratory animals	We used adult female rats (n = 5, 56 ± 5 weeks of age, mean ± sd at surgery day, four Sprague Dawley and one Long Evans, Charles -River, Sulzfeld, Germany
Wild animals	The study did not involve wild animals.
Field-collected samples	The study did not involve samples collected from the field.
Ethics oversight	All procedures were in accordance with the recommendations of the guidelines RL 2010 63 EU, Regierungspräsidium Freiburg and were approved by the Regierungspräsidium Freiburg.

Note that full information on the approval of the study protocol must also be provided in the manuscript.
